# 3D-MID Technology for Surface Modification of Polymer-Based Composites: A Comprehensive Review

**DOI:** 10.3390/polym12061408

**Published:** 2020-06-23

**Authors:** Jiratti Tengsuthiwat, Mavinkere Rangappa Sanjay, Suchart Siengchin, Catalin I. Pruncu

**Affiliations:** 1 Department of Mechanical Engineering Technology, College of Industrial Technology, King Mongkut’s of University Technology North Bangkok, Bangsue, Bangkok 10800, Thailand; joepetggs@gmail.com; 2 Natural Composites Research Group Lab, King Mongkut’s of University Technology North Bangkok, Bangsue, Bangkok 10800, Thailand; mcemrs@gmail.com; 3 Department of Mechanical and Process Engineering, The Sirindhorn International Thai German Graduate School of Engineering (TGGS), King Mongkut’s University of Technology North Bangkok, Bangsue, Bangkok 10800, Thailand; suchart.s.pe@tggs-bangkok.org; 4 Mechanical Engineering Department, University of Birmingham, Birmingham B15 2TT, UK; 5 Mechanical Engineering, Imperial College London, Exhibition Rd., London SW7 2AZ, UK

**Keywords:** 3D-MID technology, surface modification, polymer, composites

## Abstract

The three-dimensional molded interconnected device (3D-MID) has received considerable attention because of the growing demand for greater functionality and miniaturization of electronic parts. Polymer based composite are the primary choice to be used as substrate. These materials enable flexibility in production from macro to micro-MID products, high fracture toughness when subjected to mechanical loading, and they are lightweight. This survey proposes a detailed review of different types of 3D-MID modules, also presents the requirement criteria for manufacture a polymer substrate and the main surface modification techniques used to enhance the polymer substrate. The findings presented here allow to fundamentally understand the concept of 3D-MID, which can be used to manufacture a novel polymer composite substrate.

## 1. Introduction

Molded interconnected device (MID) represents the versatility of injection molding process that incorporates a conductive circuit pattern with structured metallization. It has ideal mechanical and electrical functions. This is commonly referred to as “3D-MID” and can be integrated directly into plastics structures which include the three-dimensional circuits [1,2,3,4,5].

MID technology has become well-known because of its freedom of geometric design in combination with selective structuring and metallization of 3D layout. It is able to define the angle between components, stacking of chips and foaming of cavities in contrast to the traditional printed circuit boards (PCBs) that are two-dimensional one [6,7,8,9,10,11]. Moreover, MIDs not only reduce the number of components and the cost by the embedding parts such as connector within a single device but also may save space and shorten assembly time. Therefore, MIDs are employed in numerous applications (i.e., sensor technology, medical technology, automotive, telecommunications, and antennas) [12,13,14,15].

The MID modules can be classified into class 2½D, n × 2D and 3D categories as shown in Table 1. The conventional circuit board is a flat module with 2D planar process surface. Class 2½D permits to produce sample that have flat or plane-parallel process surfaces in Z direction and on the reverse side of processed surfaces two or more plane-parallel. On the other hand, the class n × 2D and 3D is an interconnected device that consist of multiple process surfaces intersected at the angles or have freeform surfaces [16,17,18,19,20].

So far, there are reported several methods for manufacturing the MID substrate. Some of them reported the polymer substrate and surface modification in order to develop polymer-based composites. Therefore, this review presents a detailed and a meaningful insight of each processing routes. The main objective of this work is to highlight the benefits of using the MID technology for polymer-based composites by evaluating the state of the art and to provides detail of surface modification of polymer-based composites which leads to the manufacturing of a novel polymer composite substrate.

## 2. Single-Shot Injection Molding

Single-shot injection molding is preferred for high-volume production with short cycle time. Generally, the granulate of plastics are mixed and conveyed by a screw feeder from a hopper unit to the injection unit, then the liquefied plastic is injected into a mold part. Single-shot injection molding for MID technology can be sub-divided into two methods [21,22,23,24,25,26,27].

**Figure 1:**
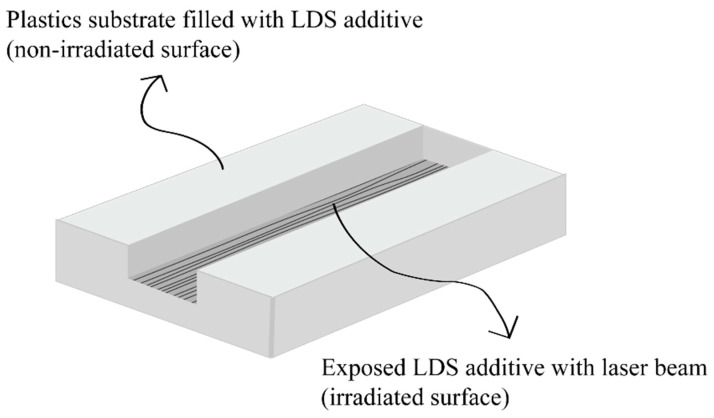
Laser ablation with simultaneous additive activation.

Laser direct structuring (LDS) was noted as an essential process for MID production over the last decades. LDS offers high level of versatility, the possibility for prototyping product with low tool costs and 3D design freedom. In order to work with LDS method, usable plastics for LDS required specific additives such as Cu_2_O, CuO or CuCl_2_, which can be added in a very high concentration to the blend during compounding step. The characteristic of these additives is extreme heat resistance and great potential to prevent nucleation during injection molding process [28,29,30,31,32,33,34,35,36,37,38]. However, it is also possible to prepare plastics for LDS without specific additives. The LDS method consists of four-step process, injection molding, laser structuring, metallization and surface finish. LDS is based on the principle of ablation and nucleation by using laser irradiation. Therefore, the laser patterning creates a microscopically rough surface and simultaneously activates the specific additives that are necessary for the metallization step [28,29,30,31,32,33,34,35,36,37,38]. The schematic of LDS method is illustrated in Figure 1.

Hot embossing is a common method used in MIDs production. It is a rapid process, clean and economical fully additive structuring that can add benefits for limited number of process steps and reduce cost of investment. In this process, a specially coated copper film is pressed into a thermoplastic substrate by a heated die with the negative conductor layout. The pressure force for embossing which is designed depends on the thickness of the copper film and substrate. There, the copper film is cut to form a positive bond to locally melted plastic close to the surface of the blank [39,40,41,42,43,44,45].

## 3. Two-Shot Injection Molding

**Figure 2:**
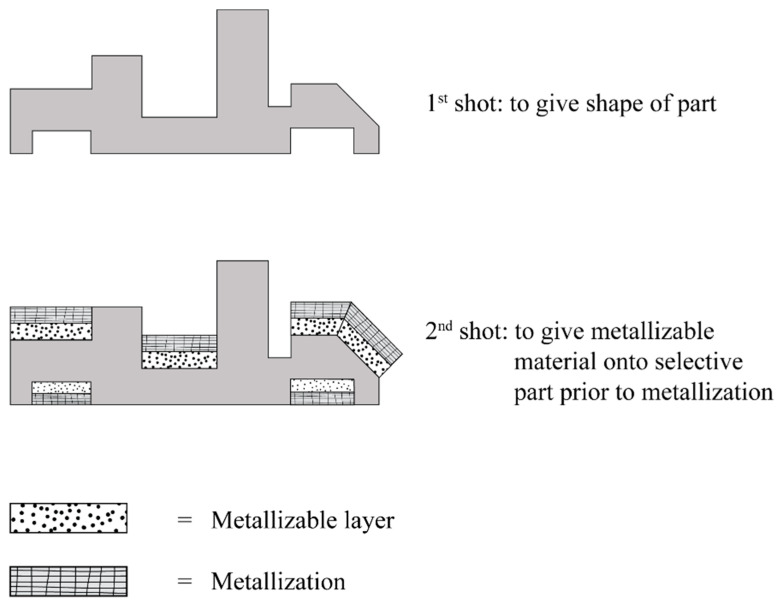
Processing step in two-shot injection molding.

Two-shot injection molding consists of two separate molding cycles. It generally uses different type of plastic that are plate-able (with plating catalyst) and non-plate able (without platting catalyst). The major advantage of two-shot injection molding is related to geometric design freedom which enable to create a very complex three-dimensional circuitry. Also, this is highly suitable for high-volume production with uniform precision. In this process, the first shot of non-plate able plastic is injected into a mold cavity to form the main component. Afterwards, the cavity in the first shot is filled by the second shot of plate able plastic on its locally surface where circuit tracks is required prior to metallization of the conductive structures [46,47,48,49,50]. The schematic of two-shot injection molding is showed in Figure 2.

## 4. Material Properties and Characteristics for MID Substrate

The substrate materials used to manufacture MID are made of plastic. The plastics not only offer more production flexibility from macro to micro-MID products but also has high fracture toughness when is subjected to mechanical loading. Moreover, they have good mechanical properties and light weight. The plastic substrates employed in the production of MID may vary depending on the manufacturer or supplier. Therefore, some important parameters must be considered when selecting the material.

- Assembly temperature
- Rheological properties
- Degradation
- Shrinkage and tolerances
- Anisotropy properties
- Tensile and flexural properties
- Metallization capability
- Electrical properties
- Environmental concerns
- Cost

In order to select a substrate material from a various type of plastics with different properties profiles, some requirement criteria of plastic materials used for MIDs are necessary. The main criteria are illustrated in Table 2 [51,52,53,54].

In order to search for more and higher capability plastic materials, there was extended the range of standard plastics and engineering plastics which includes high-specification polymers. Obviously, the requirements which apply to any base material impinge on decision-making process at several different levels. Alongside purely technical aspect such as intrinsic load ability, compatibility, the basic workability properties, ecological factors (i.e., suitability for return to the natural materials cycle) and economic factors (i.e., procurement and processing costs) influences the materials development and selection [55,56,57,58]. Therefore, the requirement outlined in Table 2 can be very diverse. In order to satisfy these requirements, the characteristics values have to line up with certain basics requirements:

- Materials have to be meaningful
- Materials have to be comparable
- Materials should be rationally measurable

The selection of the right MID substrate is often a very challenge process that should be a balance between electrical, thermal and mechanical properties and the manufacturing cost [59,60,61,62].

## 5. Thermoplastics for MID

**Figure 3:**
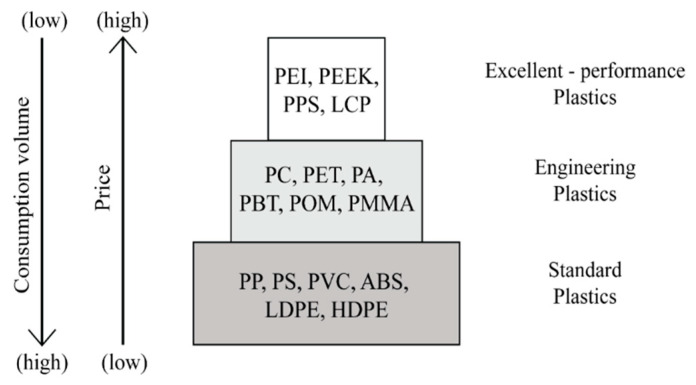
Plastics pyramid for worldwide consumption.

There are many different polymers used to manufacture the MID substrate. The choice of material and production process are primarily linked to mechanical, thermal and electrical requirement of the purpose of MID application. Thermoplastics materials are the most used to fabricate the MID substrate [63,64,65]. They can be subdivided on the basis of heat distortion resistance, long-term service temperature and the price of raw material. The plastics pyramid depicted in Figure 3 describe the three major subgroups of thermoplastic materials [66,67]. In the plastics pyramid, the presence of high-performance plastics is very scarce, but extremely interesting in terms of engineering applications. However, high-performance plastics are associated to high prices and costly processing which may entail limitations for industrial use. On the other hand, many MIDs are made of engineering-grade thermoplastics, because they are available at low cost and present virtually no difficulties for manufacturing in the existing production facilities. Typically, the characterization of an MID substrate materials depends primarily on the material properties which are responsible for indicating how the material behaves during metallization along with the chemical resistance. The pyramid overview is considered a guideline to select materials for MID [68,69].

### 5.1. Polypropylene (PP)

Polypropylene (PP) is a standard plastic that can be used in MID. It is largely nonpolar and has a crystallinity approximately 60–70%. The glass transition (T_g_) is about 0 °C and melting temperature is in the range of 155 to 160 °C thus, PP is not suitable for standard soldering process. PP has a high chemical resistance on account of its nonpolar character, and it has no inherent flame-retardant properties. Moreover, reinforced PP by glass fiber or talc can reduce the isotropic shrinkage and heat distortion resistance [70,71,72,73,74,75,76,77,78,79].

### 5.2. Syndiotactic Polystyrene (sPS)

Syndiotactic polystyrene (sPS) is partially crystalline plastic that can withstand to temperatures up to 265 °C for short periods. Long-term service temperature can reach up to 150 °C. The maximum crystallinity is approximately 52% and it depend on processing conditions. It has a melting point of 275 °C and the glass transition of 105 °C. sPS is a polymer with extremely high chemical resistance but can be easily damaged by oxidization with ozone and chorine particularly at high temperature. Moreover, sPS can be metallized by a wet-chemical or catalytic process [80,81,82,83,84,85,86,87,88,89].

### 5.3. Polyphenyl Ether (PPE)

Polyphenyl Ether (PPE) or also known as polyphenyl oxide (PPO) is an engineering plastic which is produced by blend of PPE and PS. It belongs to the group of amorphous plastics which has a good mechanical property. Its electrical properties are virtually unaffected by the temperature and frequency. The long-term service temperature is in the range of 100–110 °C. It can be chemically metallized. PPE is not only resistant to hydrocarbons but also susceptible to stress cracking on account of its styrene content [90,91,92,93,94,95,96,97,98,99].

### 5.4. Polycarbonate (PC)

Polycarbonate (PC) is an amorphous, transparent plastics that combines high strength, heat distortion resistance, and impact roughness even at low temperature. It also has good electrical insulating properties that are virtually independent of temperature and moisture. The glass transition temperature is between 140 and 150 °C [100,101,102,103,104,105,106,107,108,109].

### 5.5. Polybutylene Terephthalate (PBT)

Polybutylene terephthalate (PBT) is a partially crystalline engineering plastic. It is used primarily on account of its good dimensional stability, temperature resistance and electrical properties. PBT exhibits high resistance to many solvents, but it is not inherently flame retardant [110,111,112,113,114,115,116,117,118,119].

### 5.6. Acrylonitrile Butadiene Styrene (ABS)

Acrylonitrile butadiene styrene (ABS) belongs to the group of amorphous plastics. It exhibits good impact resistance, hardness and scratch resistance. The maximum use temperature of ABS is up to 90 °C which makes them unsuitable for standard soldering process. The glass transition temperature is about 85–100 °C. The proportion of its three constituent components can be varied which permits to adapt this material to very widely differing requirement [120,121,122,123,124,125,126,127,128,129].

### 5.7. Polyamides (PA)

Polyamides (PA) are found in an exceptionally wide range. The polyamides constituent is one of the most important groups of materials within the partially crystalline engineering plastics. Some of polyamides show properties on those of the high temperature plastics in particular PPA, PA6T/X, PA46 and PA6/6T. High mechanical loading, high damping capability, and high wear resistance are the characteristics of PA, regardless of structure. The water absorption is approximately 10%. Generally, PAs are resistant to solvents, fuels, and lubricants. However, they are not inherently flame retardant [130,131,132,133,134,135,136,137,138,139].

### 5.8. Polyphenylene Sulfide (PPS)

Polyphenylene sulfide (PPS) belongs to the group of partially crystalline and nonpolar high temperature plastics. Its structure lacks branches thus, it is highly crystalline. It is a very hard and stiff material which is suitable for process at high temperature up to 240 °C. The melting point is about 285 °C, and glass transition temperature is between 85 and 100 °C. PPS exhibits excellent chemical resistance, very low water absorption, and inherent flame resistance [140,141,142,143,144,145,146].

### 5.9. Liquid Crystal Polymer (LCPs)

Liquid crystal polymers (LCPs) consist of rigid, rod-shaped macromolecules that self-parallel in melt and form liquid crystalline structure. It can be used to manufacture extremely delicate molding with thin wall sections and long runner channels. Other characteristic properties besides high strength and rigidity in the flow direction includs the long-term service temperature of 185 to 250 °C and the melting temperature between 280 and 355 °C. LCPs are solderable with common methods and galvano-workable types also are available [147,148,149,150,151,152,153].

### 5.10. Polyetherimide (PEI)

Polyetherimide (PEI) is an amorphous high temperature plastic. It exhibits high strength rigidity and hardness even without reinforcement. The maximum long-term service temperature is about 170 °C and its glass transition temperature is 210 °C. PEI can easily solderable by reflow soldering process. It can be chemically metallized, and it is not resistant to ketones, chloroform, ethyl acetate, and methyl ethyl ketone. Moreover, it is inherently flame retardant [154,155,156,157,158,159,160].

### 5.11. Polyethersulfone (PES)

Polyethersulfone (PES) belongs to the group of amorphous, transparent and polar high temperature plastics. It is available in both reinforced and non-reinforced type. It exhibits high strength, rigidity and hardness across a wide temperature range from −100 to +200 °C. Their Tg is about 225 °C. PES can be chemically metalized by surface treatment printing and metallization under vacuum or galvanization after appropriate pretreatment process. Moreover, PES is not only chemically resistant to ketones, esters, hydrocarbons, and aromatics, but also can include highly polar solvents [161,162,163,164,165,166,167].

## 6. Modified Thermoplastics for MID

The high temperatures requirement is a changeover related to lead-free solder process imposed by the constituted law. This is the major reason why high-performance thermoplastics are virtually the only unmodified products that can be used adequately as substrate materials [168,169,170]. Engineering plastics such as PA6 and PA66 are often used as base materials on the account of their good metallization and service behaviour. Therefore, searching for approaches that are economically and relatively practically for engineering thermoplastics is still a major challenge. It is especially for assembly and connection technology process such as lead-free reflow soldering. Nowadays, these thermoplastics are enriched with up to 40% by weight of reinforcing with additive which allows to increase their mechanical limits. Glass fiber, glass balls, and a wide of variety of mineral substances are used as reinforcing additive. As far as extending their thermal limits is a concern, the MID research conducted in the recent years has brought to light two main approaches, namely electron-beam crosslinking and the use of the filler system for modifying thermoplastics, which is capable of satisfying higher requirements, particularly those deriving from the production process [171,172,173,174].

## 7. Radiation of Cross-Linked Thermoplastics

The radiation cross-linked thermoplastics is a technique which was investigated since 1950. In the past, its use was mainly restricted to the group of polyolefins. Recently, the endeavor to transfer experience gained in the radiation crosslinking to injection molding and extrusion films made of engineering plastics are a new departure into relatively unknown territory [175,176]. One of the success has been achieved by cross-linked PA6 and PBT molding. Despite their excellent mechanical and electrical properties, engineering plastics have not been adopted for high thermal load process such as reflow soldering primarily on account of their low heat distortion resistance and poor temperature resistance. This thermal weakness can be overcome by radiation crosslinking [177,178,179]. In the process of crosslinking, plastics are not only cross-linked chemically but also by radiation chemistry for example in the presence of peroxides. In principle the effect of radiation-chemical reactions can be induced by electromagnetics wave such as X-ray or gamma rays [5,180].

Radiation induced crosslinking has numerous advantages. This is because the main thermoplastics material’s properties can be improved after modified by radiation induced crosslinking as showed in Table 3 [181,182].

## 8. Thermoplastics Composites

Compounding the thermoplastics with special fillers or additives as composites materials has proven to be an effective way of integrating functions such as mechanical, thermal and electrical properties. These plastic compounds benefit from material synergies derived from the good workability of the plastics and the additional properties which are gains from the fillers. Tailoring the properties of plastic compounds such as thermally conductive in order to meet superior requirements would entail conforming with the following criteria for selection the composition of the fillers [183,184,185,186]:

- Maximization of filling
- Filler shape and size
- Filler mixtures
- Additivity

Generally, fillers such as ceramic materials are thermally conductive but electrically insulating. On the other hand, the metallic materials are thermally and electrically conductive. The high proportion of fillers can alter the behaviour radically compared to unmodified thermoplastics. On the other hand, the fracture and strain generally diminish, whereas rigidity decreases. There, the strength depends largely on the bonding of filler with the embedded matrix. Moreover, some special additives and other fillers such as glass fiber can be applied to improve the bonding between matrix and filler which result in strength and fracture strain enhancement.

Typically, the plastic compounds for MID can be manufactured from a very wide variety of fillers from the group of metallic or ceramic materials. Recently, some of fillers are coming into widespread use on account of their versatility, excellent thermal properties and because they offer considerable benefits for MID production. The preferred fillers are graphite, carbon black, copper, aluminium oxide and boron nitride. The geometry of filler is one of the main factors that influences the mechanics of reinforcement. Nowadays, they can be classified into three groups, one-dimensional filler (i.e., fibers), two-dimensional fillers (i.e., platelets) and three-dimensional fillers (i.e., powder). For one-dimensional fillers, glass fibers are widely used, because of their geometry and superior improvement in the mechanical characteristic values. The proportion of glass fiber reinforcement is generally between 15% and 50% by weight. Below 15% by weight, there is no reinforcing effect while above 50% by weight it becomes difficult to wet the fillers with the uniformity necessary for smooth surfaces. For three-dimensional filler such as glass balls usually increase only the elastic modulus. The classification of fillers by geometry is also crucial in term of thermal conductivity. Anisometric one- or two-dimensional fillers lead to higher thermal conductivities than three-dimensional fillers and spherical fillers. Therefore, the thermal conductivity is the process dependent and component dependent material property, particularly when the fillers used are anisometric. Moreover, highly filler filled, thermal conductive plastics require an adaptation of the process parameters for dependable injection molding. This is because of the higher melt viscosity and rapid cooling of the plastics melt while the mold cavity is filling. Consequently, MID design for highly filled thermally conductive compounds is always trade-off between the modified properties and workability of the materials [187,188,189,190,191].

## 9. LDS Materials for MID

The LDS is one of method used to produce MID product by using laser activation of special additive filled plastics. Therefore, the special additives that enable the LDS process must be finely distributed in body part and transferred into catalytically agent during laser activation. The requirement of these additives are good chemical compatibility and homogeneous distribution in matrix, no impairing of electrical and mechanical of substrate, excellent thermal stability and no catalytic activities in deactivated state. In this process, the preferred additives are from the group of metal oxide or mixed metal oxide especially, copper compound such as Cu_2_O, CuO or CuCl_2_. This is because its temperature resistance is extremely high thus, there no nucleation can take place while the plastic melt. LDS materials have found in the range from standard thermoplastics through engineering thermoplastics and high-performance thermoplastics which are suitable for reflow soldering process. The currently available LDS materials for industrial scale are listed in Table 4 [192,193,194,195,196,197].

## 10. Thermoset Plastic for MID

Thermoset plastic is of interest for MID substrate because it offers some high heat distortion temperature. The thermoset compound can be mixed with a wide variety of fillers, which allows to modify their properties to meet the requirements for a given applications [198,199,200,201,202]. The addition of suitable proportion of fillers can produce the coefficients of thermal expansion, heat distortion and elongation behaviour virtually identical to those of copper. Once thermoset is cured, their three-dimensional molecular network structure exhibits a very high level of dimensional stability. The important thermal properties of the current commercially available thermoset compound are listed in Table 5 [203,204,205,206].

## 11. Laser-Assisted Metallization for Polymer Materials

Regarding the MID parts, the surface properties of polymer substrate is one of the crucial factors that significantly affect metallization process, adhesion strength, and overall quality of the deposited metallic layer. Generally, the surface modification of polymer substrate can be divided into two methods, chemical and physical modification. In chemical modification, the polymer substrate is modified on selective surface by using chemical solution as solvent such as potassium manganite (KMnO_4_), nitric acid (HNO_3_), and ethyl alcohol [207,208,209]. On the other hand, laser modification is one of the popular physical surface treatment methods used in order to prepare the polymer surface for metallization. In the last decades, laser-assisted electroless metallization for polymer materials has been reported in numerous works [210,211]. Laser treatment represent a group of advanced engineering tool useful in selective modification of small surface areas of complex shapes. They have great importance in manufacturing of printed circuit boards and other small electronic devices. Laser treatment is able to change the surface geometrical structure, degradation or crosslinking of molecular chains of polymer without changing bulk properties. Laser wavelength, laser power and laser mode of operation (continuous or pulsed laser) are the factors that influences the surface properties of polymer materials. Typically, the polymers are not only absorbing well ultraviolet (UV) but also infrared (IR), whereas they are mostly transparent to visible light (Vis). Basically, UV radiation causes photolytic breaking of molecular bond within polymer materials while, the heat effects are limited. It results in the formation of free radicals that enable to initiate photochemical reactions. In the IR region, most of polymer functional groups are excited to higher vibrational and rotational energetic states that is accompanied by heat generation. The accumulation of heat is able to break the molecular bonds and thus the thermally activated reactions occur. Moreover, the polymers which are modified in the range of Vis spectrum, are commonly doped with absorbing agent (photoinitiators) which decompose and initiate various reactions. The interaction between laser irradiation and polymer are greatly depending on applied wavelength of the radiation. In the laser surface modification method, a very important parameter is energy of radiation per unit area, called laser fluence for a given polymer. The ablation of material starts when a certain value of laser fluence, the so-called ablation threshold is reached or exceeded. Therefore, the laser surface modification of materials is classified as below or above the ablation threshold [212,213,214,215,216].

## 12. Influence of Laser Irradiation on Polymer

In the laser modification of surface layer, there are two important material parameters that influence the surface properties of the polymer material. Firstly, absorption coefficient (*α*), associated with absorption of light wave energy which can be evaluated by measuring of the energy attenuation within that materials. Secondly, the refractive index (*n*), that is connected with the coefficient of reflection (*R*) of the radiation at the material-medium phase boundary [217,218]. The light wave dissipation is associated with the light absorption that can be drawn as an absorption coefficient in the following Equation (1) [219].

(1) $$\alpha =\frac{4\pi \kappa }{\lambda }$$

where *κ* is extinction coefficient and *λ* is the light wavelength.

As per Equation (1), it is clear that the light absorption coefficient of material depends on both the light wavelength and extinction coefficient. The laser radiation intensity within an examined material can be described by the empirical Beer’s law Equation (2) [220,221].

(2) $$I\left(Z\right)=\left(1-R\right)I_{0}e^{-az}$$

where *I (z)* is the laser radiation intensity within materials at a vary depth *(z)*, measured from the material surface, *R* is coefficient of light reflection from material surface, and *I*_0_ is intensity of incident laser radiation.

The coefficient of light reflection (*R*) is also an important factor for modification of the material surface layer. It can be described by Maxwell’s equation system in the case of the perpendicular incidence of light on the material surface which yields the value *R* [222,223].

(3) $$R=\frac{{\left(n-1\right)}^{2}+\kappa^{2}}{{\left(n+1\right)}^{2}+\kappa^{2}}$$

According to Equation (3), *R* value depends on both the refractive index (*n*) and the extinction coefficient (*κ*). Thus, the laser radiation intensity within material at a depth *(z)* can be rewritten by taking Equation (3) into Equation (2) which leads to following relationship [224,225] as shown in Equation (4):

(4) $$I\left(Z\right)=\left[\frac{4n+2\kappa^{2}}{\left({\left(n+1\right)}^{2}+\kappa^{2}\right\}}\right]I_{0}e^{-az}$$

## 13. The Absorption Coefficient for Laser Radiation

The polymer materials may have high absorption coefficient for laser radiation especially, in the UV range. Penetration depth (1/*α*) of laser light at 147 nm for example, polyethylene (PE) is about 34 nm, whereas its gamma radiation is about 15 cm [226]. This implies that the energy is absorbed in a thin surface layer of polymeric material irradiated even by using ultraviolet radiation. In addition, the surface layer properties of polymeric materials also related to the laser ablation. Basically, the laser ablation is based on physicochemical changes induced by the laser radiation and resulted in tearing off fragments of the irradiated surface layer in the ablation process [227,228,229,230,231]. In the laser ablation of polymeric materials, there are two main process, photochemical and photothermal that may proceed simultaneously. The photochemical ablation consists in photolytic breaking of chemical bond. It was done by electron excitation in macromolecule segments to high-energy electron states. The essential phase of the ablation process begins when a large number of chemical bonds simultaneously undergo breaking upon irradiation by high-energy laser pulses [232]. The ablation threshold (*E_th_*), and the number of broken chemical bonds (*n*) can be drawn as in Equation (5) [233,234].

(5) $$E_{th}=n\left[\frac{h\nu }{\phi \alpha \left(1-R\right)}\right]$$

where, *R* is coefficient of laser radiation reflection of the polymeric surface, *φ* is the quantum yield of bond breaking (0 to 1), *α* is coefficient of radiation absorption, and *hν* is photon energy. On the other hand, in the photothermal ablation process is also assumed that the laser radiation strongly is absorbed by the material excites molecules to high-energy state. Due to mutual collisions, the molecules relax from this state to ground state thus, heat generated in this way causes an increase in temperature being sufficient for breaking of chemical bonds in polymeric materials [235]. When the material temperature exceeds a certain value, called threshold ablation temperature (*T_D_*), the process of thermal ablation of polymeric materials begins. The relationship between *E_th_* and *T_D_* can be expressed as in Equation (6) [236,237].

(6) $$E_{th}=Cw\left[\frac{T_{D}-T_{R}}{\alpha \left(1-R\right)}\right]$$

where *T_R_* and *C_w_* are the initial temperature and specific heat of polymeric materials, respectively.

## 14. Photochemical and Photothermal Ablation

**Figure 4:**
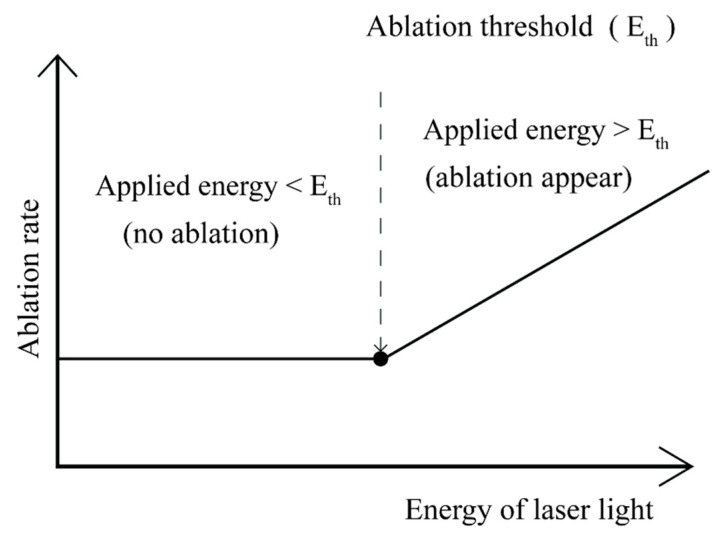
Laser ablation rate as a function of a unit energy of laser light.

Photochemical and photothermal ablation proceed works simultaneously and are difficult to separate. The thickness of material fragment torn off the surface layer i.e., ablation depth, depend on both photochemical and photothermal ablation mechanisms. Hence, the ablation depth (*L*) of materials can be expressed as a sum of the two components as presented in Equations (7) and (8) [238,239,240].

(7) $$L=\frac{1}{\alpha_{eff}}\ln\left(\frac{E_{j}}{E_{th}}\right)+Aexp\left(\frac{-E_{a}}{\kappa_{B}T}\right)$$

(8) $$L=L_{chem}+L_{therm}$$

where *E_j_* is the energy per laser pulse, *E_a_* is activation energy for ablation, and *α_eff_* is effective coefficient of absorption. The first component in Equation (7) represents the photochemical ablation model which is derived from the Beer’s law, whereas the second component represents the photothermal ablation model based on the Arrhenius’s law associated to Equation (8) [241,242,243,244]. The ablation rate of the surface layer of polymeric materials is illustrated in Figure 4 [245,246]. The ablation rate is defined as a quotient of the total ablation depth (*L*) and the number of laser pulses (*N*) as a function of *E_j_* when is neglected the thermal component. The rapid tearing off fragments of polymeric materials starts when the laser pulse energy exceeds the ablation threshold. During that time, some changes in surface geometric structure appear while no significant chemical change occur. Moreover, the ablation rate depends also on the nature of material to be modified, *E_j_* value, laser radiation wavelength, laser pulse duration (pulse width) and ambient condition. Because so many variable factors, including the two-step absorption and attenuation of radiation beam by the material being torn off, there are a number of models for the mechanism of the ablation process [247,248,249,250,251].

## 15. Selection Criteria of Laser for Polymers

There are many factors that affect surface layer properties such as absorption coefficient, laser beam angle of incidence, and energy distribution of laser beam. Therefore, a suitable criterion for the selection of laser for polymer is required. For instance, the coefficients of absorption of the laser UV radiation for poly(ethylene terephthalate) (PET) and polystyrene (PS) are significantly higher than those for polyethylene (PE) and polypropylene (PP). PET and PS absorb the laser UV radiation mostly in the surface layer of thickness not exceeding 0.1 and 0.4 micrometer, respectively. This is in contrast to PP and PE which the absorption occurs within much thicker layer. Some of additives such as polyolefin and benzophenone are being used for the improvement of UV and UV-Vis light absorption [252,253,254,255]. The specific heat and thermal diffusion constant of the polymeric material can be a concern because they are related to minimization of thermal damages into material. In the recent years, the number of lasers operating with different ranges of wavelength and various mode (pulsed or continuous) are rapidly growing. They can be classified according to various criteria. One of the most important criteria is the type of active medium or gain medium. Basically, there are three types of active medium which are used in present [256,257]:

- Gas laser (excimer laser, nitrogen or carbon dioxide laser)
- Solid-state lasers (neodymium, Nd:YAG laser)
- Dye laser

Table 6 presents a summary with the list of available lasers and their capacity [258,259].

## 16. Metallization

In order to create complete MID parts integrated within the circuit track, plastic parts must be coated with metal ion by a process called metallization. The metallization or perhaps called plating is a process to plate or deposit a metal ion (i.e., Cu^2+^, Ni^2+^) into the conductive or non-conductive substrate. The goal of this process is to enhance the specific properties of plastics including reflectivity, abrasion resistance, and electrical conductivity. Therefore, plating on plastics has received considerable attention in the manufacturing of printed circuit boards (PCBs), automotive parts and electromagnetic interference (EMI) shielding applications [260,261,262,263]. Over the last decades, the plastics was successfully metallized with gold, silver, nickel and copper [264,265]. Generally, metallization of plastics is classified into two groups that are primary and secondary metallization. In primary metallization, plastics can be done by depositing a thin layer of metal having thickness approximately 10–50 µm. On the other hand, the secondary metallization is conducted after primary metalized parts to increase the thickness of metallic layer up to 180 µm or above. There are many methods for the metallization of plastics as following [266,267]

- Dipping in a metal paint
- Sputtering
- Vapour deposition technique
- Electro plating
- Electroless plating

## 17. Electro and Electroless Plating

The electro and electroless plating are the most important methods for producing functional and decorative finishes in the requirement for MID applications. Both electro and electroless represent a chemical reduction between the reducing agent, in the presence of solution, and the metal ion. During the plating process, a thin layer of catalyst is applied to localized surface. For instance, a layer of metal (i.e., copper or nickel) is plated as a result of the metallic phase that appears on the solid surface. For the electro plating or perhaps called galvanic plating, it is necessary to use battery or rectifier. The applied electric current is combined with a chemical solution to reduce the metal cations. Basically, there are two main components, the parts that is plated called cathode and the metal which is plated on the parts is called anode. On the other hand, electroless plating, also known as autocatalytic plating, is a process which uses purely chemical reduction process without any electrical energy dispersal [268,269,270,271,272,273]. It is possible to obtain metalize on dielectric surface at ambient temperatures by using some simple aqueous solutions. Therefore, electroless plating is widely used in modifying the surface of plastics. Especially it was noted a significant growth on the printed circuit market. The schematic of electro and electroless plating are illustrated in graphical abstract, and the various advantageous and disadvantageous between electro and electroless plating are presented in Table 7 [274,275,276].

## 18. Electroless Plating Procedure

Electroless plating on plastic is a technologically and chemically complex process. Generally, electroless plating consists of the following steps [277,278,279,280,281].

(i) Cleaning step:

This step is not only used for cleaning the surface of plastics to remove oil, dirt but also used to produce roughness which leads on improving surface area. The cleaner typically belongs to alkaline group which include neutral or acidic materials. The most important factors for a superior cleaning solution are: temperature of cleaner, concentration of cleaner and cleanliness of cleaner after cleaning.

(ii) Etching steps:

Etching is the key stage in plating process for achieving a good metal-plastic bonding. The employed surface is not only chemically etched but also physically etched resulting in the development of pores that can increase the surface area thereby providing the opportunity for superior contact between metallic layer of plating. During the chemical etching, the plastic parts are immersed in an oxidant solution which is either chromic acid in aqueous sulphuric acid or hydrofluoric acid containing sulphuric acid. It is applied on the selective surfaces to be plated. The etching time is normally for 5–15 min with temperature in the range of 60–65 °C. In addition, a laser can be applied for physically etching the plastic parts. The quality of the etching surface depends on laser mode (pulse or continuous), laser wavelength, and energy of laser beam.

(iii) Neutralization steps:

Neutralization is necessary for removing the residual of oxidant that remains during the etching steps. The reducing agent such as ferrous ions aids to prevent the inhibition of the catalyst. This is because even trace may completely inhibit electroless deposition of metallic layer on the plastic surface.

(iv) Activation steps:

In this process, the modified surface is contacted with an activator or catalysts usually in colloidal suspension as catalyst powder. The catalyst deposited in the surface micro-cavities is formed during conditioning for subsequently the electroless plating. The activation process is typically carried out at 40–45 °C for 3–5 min. Higher concentration of activator or too long immersion time lead to improper activation which probably cause poor metallic bonding. There are many commercial catalysts that can be used in the activation process as showed in Table 8 [282,283,284,285]. Generally, the electroless plating is regarded as a dehydrogenation reaction since the hydrogen may develops simultaneously with metal reduction. According to Table 8, palladium (Pd) is the most active from the catalyst list. It is because its exchange current density for hydrogen gas evolution reaction (−log i_0_) is the lowest. However, palladium is unable to form itself on the plastics surface. Therefore, the combination of stannous (Sn) with palladium is preferred because stannous is an excellent reducing agent for palladium when is used for coating of non-conductive surface. Stannous used in form of stannous chloride serves to wet the surface. The uniform layer of reducing agent afterward is subjected to intermediate water rinse which then is converted into insoluble, hydrous stannous oxychloride coating. When palladium chloride solution is dipping in, the palladium chloride may be reduced to metallic palladium (Pd^0^) and bound to the desired surface. Moreover, the concentration of palladium and stannous is normally 5 × 10^−6^ g/cm^2^ and 20 × 10^−6^ g/cm^2^ respectively, which usually is used for plating of plastics [286,287,288,289].

(v) Acceleration steps:

The acceleration is a process which permits to activate the catalyst and during the activation step the activity is more intense. The accelerator can be acidic or alkaline solution. The activated surface is typically washed and immersed in an acceleration bath at 30–35 °C for 3–5 min. There palladium and stannous mat be used as catalysts too. The excess of stannous on the activated surface allows stabilizing stannous ions by the accelerator solution consequently, the Pd^2^^+^ as a catalyst is reacted and formed into Pd^0^. The chemical reduction of palladium (II) in the presence of stannous (II) after acceleration can be drawn by [290,291,292,293,294,295]:

(9) $${\mathrm{Pd}}^{2+}+{\mathrm{Sn}}^{2+}\overset{\mathrm{HCl}}{\to }{\mathrm{Pd}}^{0}+{\mathrm{Sn}}^{4+}$$

(vi) Deposition or plating steps:

Deposition or plating is the final process for electroless metallization. In this stage, the activated surface which is prepared throughout the solution usually contains metal-salts as a reducing agent. The plating layer forms by oxidation-reduction reaction on the activated surface. Electroless plating bath typically contains metal-salts, reducing agent (i.e., formaldehyde), alkaline hydroxide (i.e sodium hydroxide), chelating agents (i.e., EDTA, Rochelle salts), stabilizer and brightener. This process is conducted at bath temperature in the range 45–65 °C [296,297,298,299]. A versatile explanation of mechanism of electroless plating process based on electrochemical reactions is presented [300,301,302]. The reducing agents are anodically oxidized on the catalyst surface while the electrons obtained are transferred to metal ions by a cathodically reduction. An excellent example is electroless plating of copper. The copper ions are reduced by formaldehyde process at room temperature (30–35 °C) in alkaline solutions (pH ≅ 12–14). At this stage copper ions are bounded into a complex. The most suitable Cu^2+^ ligands for electroless copper plating solutions contain polyhydroxy compounds from tertiary amine groups and hydroxy groups in most common practice used K-Na tartrate, Na_2_EDTA, NaOH and formaldehyde. The chemical reduction for copper plating is described by [303,304,305]:

2CH_2_O + 40H^−^ → Cu^0^ + 2HCOO^−^ + H_2_ + 2H_2_O (10)

The measurement of polarization resistance (*R_p_*) is a method that can provide information about the mechanism of plating process. The polarization resistance is inversely proportional to the process rate (*i*). Here, the relationship between *R_p_* and *i* is written as described in Equations (11) and (12) [306,307,308], respectively.

(11) $$i=\frac{b_{a}b_{c}}{R_{p}\left(b_{a}+b_{c}\right)}$$

(12) $$R_{P}={\left(\frac{dE}{di}\right)}_{i=0}$$

where *b_a_* and *b_c_* are Tafel coefficients (*b* ≅ 1/*αnf*), *α* is the transfer coefficient, *n* is the number of electrons involved in the reaction for one molecule of reactant and *f* is equal to *F*/*RT* (*F =* Faraday number).

Another factor which should be considered during plating process is deposition rate. Deposition rate is expressed in unit of micrometer per hour. When the concentration of reducing substance is not maintained at a constant level, the deposition rate will start to decrease. The deposition rate is often given as average rates that depend on the ratio of the surface to be plated and the volume of solutions (dm^2^/L). The dependence of deposition rate (*v*) for the specific concentration of reducing substance is described by an empirical equation as follow [309,310,311,312,313,314]:

(13) $$v=k{\left[{Me}^{n+}\right]}^{a}{\left[Red\right]}^{b}{\left[H^{+}\right]}^{c}{\left[L\right]}^{d}$$

where *k* is the rate constant, *L* is the concentration of free ligand; *Me^n+^* and *Red*, are metal ions and reducing agent respectively. The exponents *a* and *b* are usually smaller than unity, *c* is a negative value and *d* is usually close to zero when the ligand is substituted. However, this relationship is for a general case. There, the electroless deposition rate is normally about 2–5 µm/h.

## 19. Future Recommendation

The strategies for the development of polymer-based composites undoubtedly strive to address the surface modification and selection of catalytic activity within nowadays challenging metals in order to achieve the requirement of MID technology. The surface modification is paramount important to address this challenge. However, only a catalytic activity can offer a simultaneous effect to all modification techniques. Accordingly, a possible solution forward to accelerating the research and development of polymer-based composites for MID, is to bridge the research gap between surface modification techniques of polymer-based composites and catalytic activity of metals agent. However, the efficiency of chemical modification surface is limited due to its chemical reaction.

On the other hand, laser-assisted modifications have successfully embarked to modify polymer surface. Similarly, a number of fabrications of electroless plating solution with palladium have been reported. Hence, the way forward, is to produce a combination of laser-assisted modification and electroless plating solution which incorporated palladium. This can extend the scope of polymer-based composites and MID technology to shorten the gap between lab scale research and industrial applications in future.

## 20. Conclusions

The higher requirements of three-dimensional electronic parts associated to better functioning and continuum miniaturization have encouraged the scientific community to focus their research and develop novel method such as 3D-MID technology. The 3D-MID represents the versatility of injection molding process that incorporates a conductive circuit pattern with structured metallization. It can reach ideal mechanical and electrical functions and can be effectively integrated directly into the plastics substrate which include the three-dimensional circuits for automotive parts, mobile phones, and medical devices. This review was devoted to providing a robust understanding of 3D-MID modules, to present the requirement criteria for manufacture polymer substrate, and the main surface modification techniques used to enhance the polymer substrate. The findings presented here allows to fundamentally understand the concept of 3D-MID which can be used to create novel polymer composite substrate. The importance of selection polymer substrate including surface modification, which play a crucial role was emphasized. By applying an appropriate laser treatment on a polymer substrate, it will be possible to obtain better surface characteristics which enable to improve the metallization process. The laser treatment applied to a polymer substrate could be considered a very versatile technique because its effectiveness in surface modification permits the use of polymer composites within multiple critical applications.

## Figures and Tables

**Table 1:** The classification of MID modules.

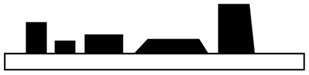	The conventional planar process surface (2D)
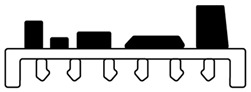	The planar process surface with 3D element on the process side (2½D)
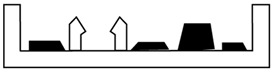	The planar process surface with 3D element on the opposite process side (2½D)
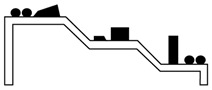	The multi parallel plane process surfaces (2½D)
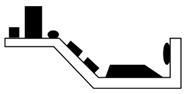	The multi process surfaces with different angles (2D x n)
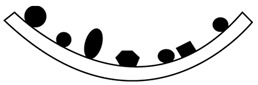	The regular cylindrical process surfaces (3D)
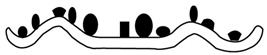	The freeform process surfaces (3D)

**Table 2:** Requirement criteria for polymer materials used in MIDs.

Thermal properties	- Heat distortion resistance - Thermal expansion - Melting and crystallization
Mechanical properties	- Strength and yield strength - Fracture elongation - Stiffness (E modulus)
Electrical properties	- Dielectricity - Electrical puncture resistance
Workability properties	- Flowability - Shrinkage - Distortion
Compatibility	- Plastic/plastic - Plastic/metal
Environmental compatibility	- Recycling - Scarcity

**Table 3:** Improvement in thermoplastics properties by radiation induced crosslinking.

Mechanical Properties	Thermal Properties	Chemical/Physical Properties
- Strength - Moduli - Abrasion resistance - Creep behavior	- Temperature resistance - Reduction in thermal expansion - Solderability - Flame retardation	- Chemical resistance - Reduction in solubility - Increase adhesion - Hydrolysis resistance

**Table 4:** The list of LDS materials.

Polymer Matrix	Supplier Company	Commercial Grade Name
ABS	RTP	699 X 113386 B
	Trinseo	MAGNUM LDS/ABS 3453
PC	RTP	399 X 113385 B
	MEP	XANTAR LDS 3750
	Blustar Chengrand	SUNPLAS LDS C0040
	Trinseo	EMERGE LDS/PC 8900
	Kingfa	Vismid SOL 2100 LDS
PA/PPA	BASF	Ultramid T4381 LDS
	DSM	ForTii LDS 85
	EMS	Grilamid 1SBVX-50H LDS
	Evonik	Vestamid HT plus LDS 1031
	Kingfa	Vismid SOL 65250 LDS
PA/PPA	RTP	RTP 299 X 113399 H
	MEP	Reny XHP 1351L
PBT	RTP	1099 X 127271 C
	Evonik	Vestodur X9423
PPE	Premix	Preperm 260 LDS
LCP	RTP	RTP 3499-3 X 113393 A
	Ticona	Vectra E840i LDS
PEI	RTP	2199 X 127272 A
PPS	DIC	LP-150-LDS

**Table 5:** List of current commercial thermoset compound and thermal properties.

Polymer Name	Elongation (10^−6^/K) (ISO 11359)	Heat Distortion Temperature (°C) HDT-A (1.8 N/mm^2^) (ISO 75-2)
Phenolic resin	16–24	>250
Epoxy resin	15–25	>250
UP resin	10–20	>250
DAP resin	10–20	>250

**Table 6:** The list of available lasers in industry.

Wavelength (nm)	Active Medium	Operation Mode	Average Power (W)
193	ArF excimer	pulsed mode	1–100
248	KrF excimer	pulsed mode	1–100
308	XeCl excimer	pulsed mode	1–100
351	XeF excimer	pulsed mode	1–100
337	N_2_	pulsed mode	0.1
351–1092	Ar^+^	pulsed/continuous	0.001–0.1
262/355/532/1064	Nd:YAG	pulsed/continuous	100
697	Al_2_O_3_ (ruby laser)	pulsed/continuous	1
9000–11,000	CO_2_	pulsed/continuous	10,000
808/940/980	GaAs	continuous mode	1000

**Table 7:** Electro and electroless various advantageous and disadvantageous.

Electro Plating	Electroless Plating
Plating thickness: 40–50 µm	Plating thickness 8–10 µm
Controlled electro plating reaction	Complicated chemical process
Electrical conduction	No conduction problem
High quality surface finish	Rough surface finish

**Table 8:** Comparison of catalytic activity of metals.

Metal	−log i_0_ (A/cm^2^) (Exchange Current Density for Hydrogen Evolution Reaction)
Palladium	3.0
Platinum	3.1
Rhodium	3.6
Iridium	3.7
Nickel	5.2
Gold	5.4
Silver	5.9
Copper	6.3

## Nomenclature

T_g_	Glass transition temperature [°C]
*α*	Laser absorption coefficient
*α_eff_*	Effective coefficient of laser absorption
*n*	Reflective index
*R*	Coefficient of reflection
*R_p_*	Polarization resistance [Ω/cm^2^]
*κ*	Extinction coefficient
*λ*	Light wavelength [nm]
*I*	Laser radiation intensity [W/sr]
*I*_0_	Intensity of incident laser radiation [W/sr]
*i*	Plating process rate [s]
*E_th_*	Ablation threshold [J/cm^2^]
*E_j_*	Energy per laser pulse [J/cm^2^]
*E_a_*	Activation energy for ablation [J/cm^2^]
*φ*	Quantum yield of bond breaking (0 to 1)
*hν*	Photon energy [eV]
*T_D_*	Threshold ablation temperature [°K]
*T_R_*	Initial temperature [°K]
*C_w_*	Specific heat of polymeric materials [J/kg·K]
*L*	Ablation depth [mm]
*L_chem_*	Photochemical ablation depth [mm]
*L_therm_*	Photothermal ablation depth [mm]
*b_a_*, *b_c_*	Tafel equation coefficients
*v*	Dependence of deposition rate [dm^2^/L]
